# PEGS: An efficient tool for gene set enrichment within defined sets of genomic intervals

**DOI:** 10.12688/f1000research.53926.1

**Published:** 2021-07-15

**Authors:** Peter Briggs, A. Louise Hunter, Shen-hsi Yang, Andrew D. Sharrocks, Mudassar Iqbal

**Affiliations:** 1 Bioinformatics Core Facility, Faculty of Biology, Medicine and Health, University of Manchester, Manchester, M13 9PL, UK; 2 Division of Diabetes, Endocrinology & Gastroenterology, Faculty of Biology, Medicine and Health, University of Manchester, Manchester, M13 9PL, UK; 3 Division of Molecular & Cellular Function, Faculty of Biology, Medicine and Health, University of Manchester, Manchester, M13 9PL, UK; 4 Division of Informatics, Imaging & Data Sciences, Faculty of Biology, Medicine and Health, University of Manchester, Manchester, M13 9PL, UK

**Keywords:** Genomic data integration, ChIP-seq, RNA-seq, gene set enrichment, genomic intervals

## Abstract

Many biological studies of transcriptional control mechanisms produce lists of genes and non-coding genomic intervals from corresponding gene expression and epigenomic assays. In higher organisms, such as eukaryotes, genes may be regulated by distal elements, with these elements lying 10s–100s of kilobases away from a gene transcription start site. To gain insight into these distal regulatory mechanisms, it is important to determine comparative enrichment of genes of interest in relation to genomic regions of interest, and to be able to do so at a range of distances. Existing bioinformatics tools can annotate genomic regions to nearest known genes, or look for transcription factor binding sites in relation to gene transcription start sites. Here, we present PEGS ( Peak set Enrichment in Gene Sets). This tool efficiently provides an exploratory analysis by calculating enrichment of multiple gene sets, associated with multiple non-coding elements (peak sets), at multiple genomic distances, and within topologically associated domains. We apply PEGS to gene sets derived from gene expression studies, and genomic intervals from corresponding ChIP-seq and ATAC-seq experiments to derive biologically meaningful results. We also demonstrate an extended application to tissue-specific gene sets and publicly available GWAS data, to find enrichment of sleep trait associated SNPs in relation to tissue-specific gene expression profiles.

## Introduction

Gene expression control in higher organisms is achieved through a complex hierarchical process involving opening of chromatin, histone modifications, and binding of transcription factors (TFs). Experimental approaches to understand transcriptional regulatory mechanisms in a biological context involve large-scale measurement of gene expression. Depending on the design of the experiment, these analyses produce differentially expressed gene sets or clusters for further analysis. These studies are often complemented by assays which map, on a genome-wide scale, TF binding sites (ChIP-seq) or regions of chromatin accessibility (DNase-seq, ATAC-seq). Analyses of these data produce a collection of genomic intervals (peak sets). An important computational task is then to integrate these data to produce meaningful results; i.e. to relate gene sets to peak sets to aid functional interpretation. Bearing in mind distal regulation, an important consideration here is to be able to calculate gene set enrichment at multiple genomic distances from peak sets, and to be able to do this efficiently within the same analysis.

We present a new tool – PEGS ( Peak set Enrichment in Gene Sets) ^1^ – which calculates mutual enrichment of multiple gene sets associated with multiple peak sets, simultaneously and efficiently. This can be at user-defined peak-to-TSS (transcription start site) distances, as well as constraining to topologically associated domains (TADs). Thus, PEGS quickly produces an overall picture of gene set enrichment in relation to peaks, and shows at what genomic distances this is most pronounced. It is applicable to gene sets derived from any source, and peak sets derived from different epigenomic assays, as well as single nucleotide polymorphisms (SNPs) from genome-wide association studies (GWAS).

## Methods

### Architecture and Implementation

**Figure 1.**
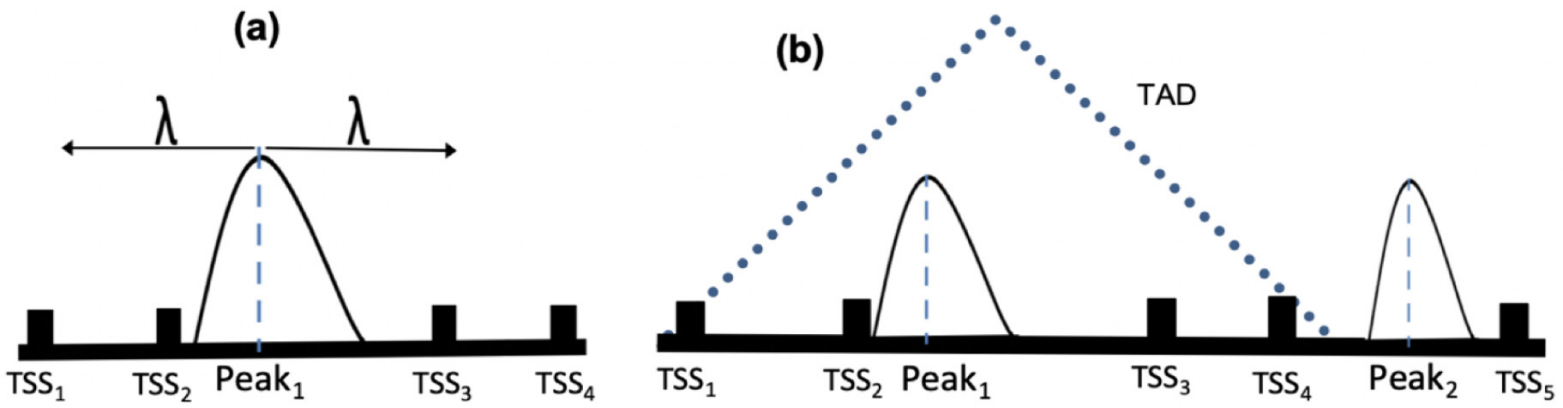
Cartoon showing peak expansion and overlapping TSSs in PEGS, with a specified genomic distance *λ* from the centre of the peak in both directions ( **a**) where *TSS*_2_ and *TSS*_3_ are included and a TAD overlapping with the left peak in ( **b**) where all four TSSs within the TAD are included

In PEGS, input peaks are extended in both directions for a given distance or constrained within known TAD boundaries, if provided ( Figure 1). Subsequently, the enrichment of the input gene set is calculated among the genes whose TSSs overlap with the extended peaks. These tasks are performed in PEGS as follows:

1. Creating a gene interval file in BED (Browser Extensible Data) format for all genes in the given genome using *refGene* from UCSC Table Browser. This reference TSSs BED file only needs to be created once (human hg38 and mouse mm10 are provided with the tool; a utility is provided to create these for other genomes).
2. For a given peak set, peaks are extended to specified genomic distances in both directions, and up to overlapping TAD boundaries. Intersection of these extended peaks with the gene intervals BED file from step 1 is calculated using BEDTools (RRID:SCR_006646) ^2^, leading to a gene set whose TSSs overlap with extended peaks.
3. Using the intersection of the input gene set, and unique genes from step 2, a Hypergeometric test is performed to calculate the p-value using Equation 1, similar to GREAT (RRID:SCR_00580) ^3^. Here, *M* is the total number of genes in the genome, *N*_c_ is the number of genes in the input cluster/set *c*, *N*_p_ is the number of unique genes overlapping the peaks for given distance and *n _pc_* is the intersection of two gene sets.

$$p\;-\;value\;=\;\sum_{x=n_{pc}}^{min(N_{p},N_{c})}\frac{\left(\begin{array}{c} N_{p}\\ x \end{array}\right)\;\left(\begin{array}{c} \left(M-N_{p}\right)\\ \left(N_{c}-x\right) \end{array}\right)}{\left(\begin{array}{c} M\\ N_{c} \end{array}\right)}\;(1)$$

Step 2 and 3 are repeated for every combination of gene cluster, peak set and distance and/or TADs. The final combined heatmap shows − *log*_10_ of the resulting p-values.

PEGS is implemented in Python 3, where we have reused functions from existing Python packages included with Python distributions, or available from the Python Package Index (PyPI). We also make use of BEDTools ^2^ for working with genomic intervals. We provide online documentation ( https://pegs.readthedocs.io/en/latest/), and an example analysis with input data at the PEGS GitHub repository. 

### Operation

PEGS works with Python >= 3.6 and, when installed through pip, automatically installs all the dependencies. These are listed in *requirements.txt* file in our PEGS GitHub repository. We provide extensive documentation online at https://pegs.readthedocs.io which includes easy-to-follow instructions about:

- Installation and system requirements
- Format of input files, output files, and graphics
- PEGS commands for standard operations, as well as running PEGS with additional input options, e.g. TAD definition files
- Creating customised reference TSSs files for new genomes

## Results

### Use Cases

**Figure 2.**
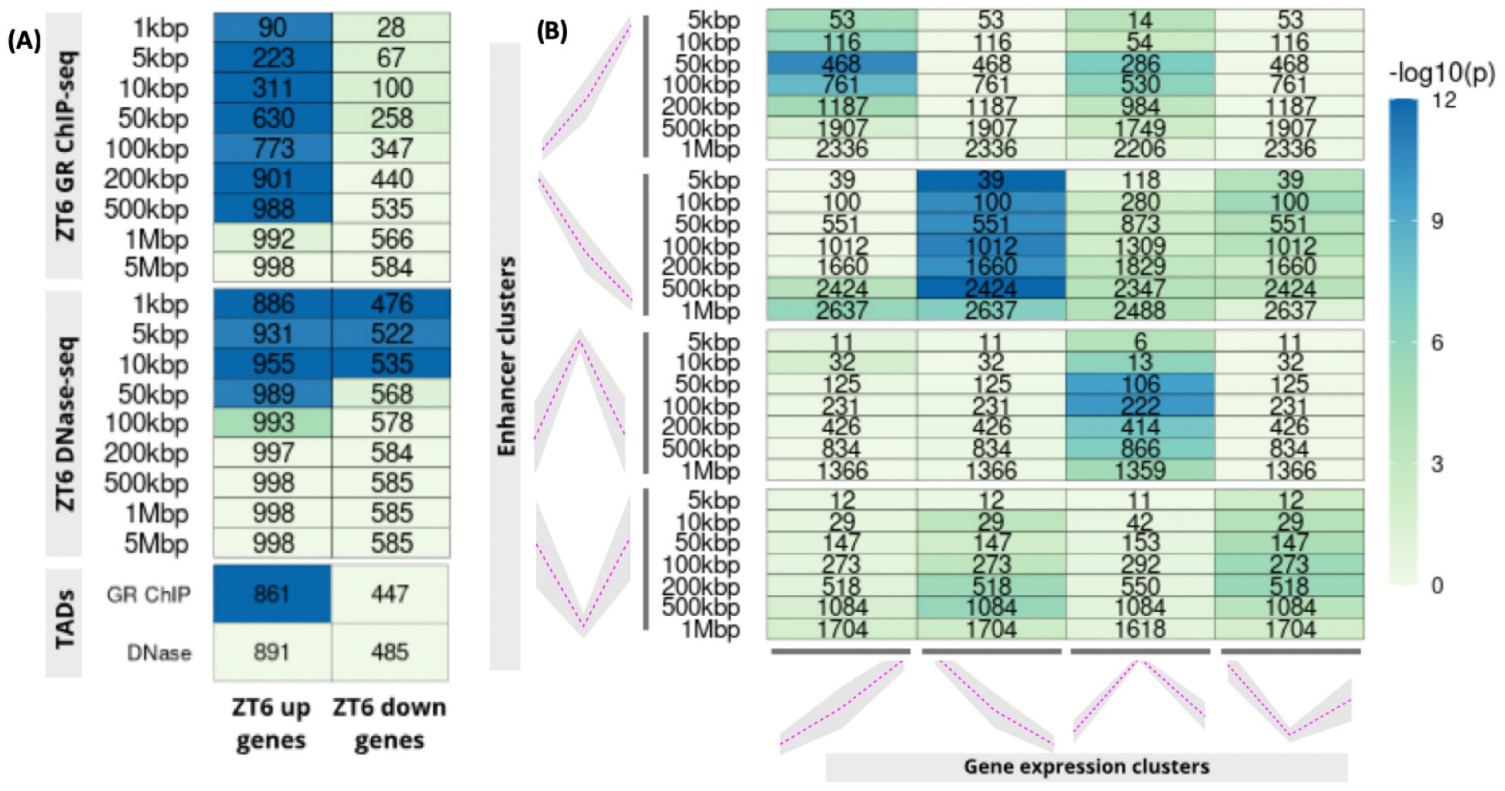
PEGS applications: ( **A**) gene expression, ChIP-seq and DNase I data on mouse liver, ( **B**) gene clusters derived from scRNA-seq and intergenic putative enhancer clusters from bulk ATAC-seq from three matching early stem cell differentiation time-points. In both plots, numbers in the cells show common genes among the input genes (x-axis) and genes overlapping with expanded peaks (y-axis) and the colour shows − *log*_10_ of p-value (Hypergeometric test)

Here, we present three use cases where we apply PEGS to different publicly available data sets. The format of input files is the same for all use cases below. Gene clusters are provided as text files with one gene symbol on each line; genomic region coordinates are provided in standard BED format. These input files for Use Case 1, as well as example analysis reproducing Figure 2A, are provided in our GitHub repository ( https://github.com/fls-bioinformatics-core/pegs). 

### Use Case 1: Application of PEGS provides insight into glucocorticoid-mediated gene regulation in mouse liver

The first application ( Figure 2A) uses up- and down-regulated glucocorticoid target genes obtained by an RNA-seq study of liver samples from mice treated acutely with synthetic glucocorticoid dexamethasone or vehicle ^4^. Corresponding GR ChIP-seq and chromatin accessibility data (DNase I hypersensitive (DHS) regions) were obtained from 5, and 6 respectively, whilst the mouse liver TAD boundaries were obtained from 7. Raw published datasets were downloaded from GEO Sequence Read Archive (RRID:SCR_005012) using *sratoolkit* v2.9.2 ( http://ncbi.github.io/sra-tools/). Reads were aligned to the reference genome (mouse *mm10*), sorted and indexed using *Bowtie2* (v2.3.4.3, RRID:SCR_005476, ^8^) and SAMtools (v1.9, RRID:SCR_002105, ^9^). MACS2 (v2.1.1.20160309, RRID:SCR_013291, ^10^) was used to call peaks, using default settings. PEGS analysis shows strong association of dexamethasone up-regulated genes with dexamethasone-induced GR peaks at distances up to 500kbp from these peaks, but no enrichment of down-regulated genes, indicating distinct mechanisms of gene activation and repression by glucocorticoids. At the same time, there is promoter proximal enrichment for both up- and down-regulated genes in the DHS regions.

### Use Case 2: PEGS demonstrates association of differential chromatin accessibility and gene expression during embryonic stem cell differentiation

Next, using PEGS, we calculated enrichment of gene clusters derived from single-cell RNA-seq and open chromatin regions defined by bulk ATAC-seq at three matching stages (ESCs- embryonic stem cells, day1 EpiLCs - epiblast-like cells, day2 EpiLCs) of early embryonic stem cell differentiation ^11^. The intergenic regions (peak sets) were defined as those with differential accessibility between any two time points and were clustered into four profiles based on z-score of tag densities, as described in 11. Similarly, differentially expressed genes were identified from pseudo-bulk gene expression data at each time point, and were clustered into four patterns. As shown in Figure 2B, strong association is observed between the matching gene expression (x-axis) and chromatin opening profiles (y-axis) at intergenic enhancers, reflecting correspondence between differential accessibility and gene expression changes.

### Use Case 3: Extended application: PEGS detects enrichment of sleep trait SNPs in tissue-specific genes

Furthermore, we present an extended application of PEGS to GWAS data and find associations of SNPs for different sleep phenotypes with sets of tissue-specific genes from the Genotype-Tissue Expression ( GTEx) Portal, RRID:SCR_013042). For this purpose, we downloaded GWAS data from the Sleep Disorder Knowledge Portal (RRID:SCR_016611) for certain sleep associated phenotypes (with genome wide p-value cutoff <=5 *e* − 8) and calculated enrichment of tissue-specific gene lists defined using the GTEx Portal. Using median transcripts per million (TPM) data for different tissues in GTEx, a gene list for a tissue was defined as genes with 5x median TPM compared to the average in the remaining tissues.

**Figure 3.**
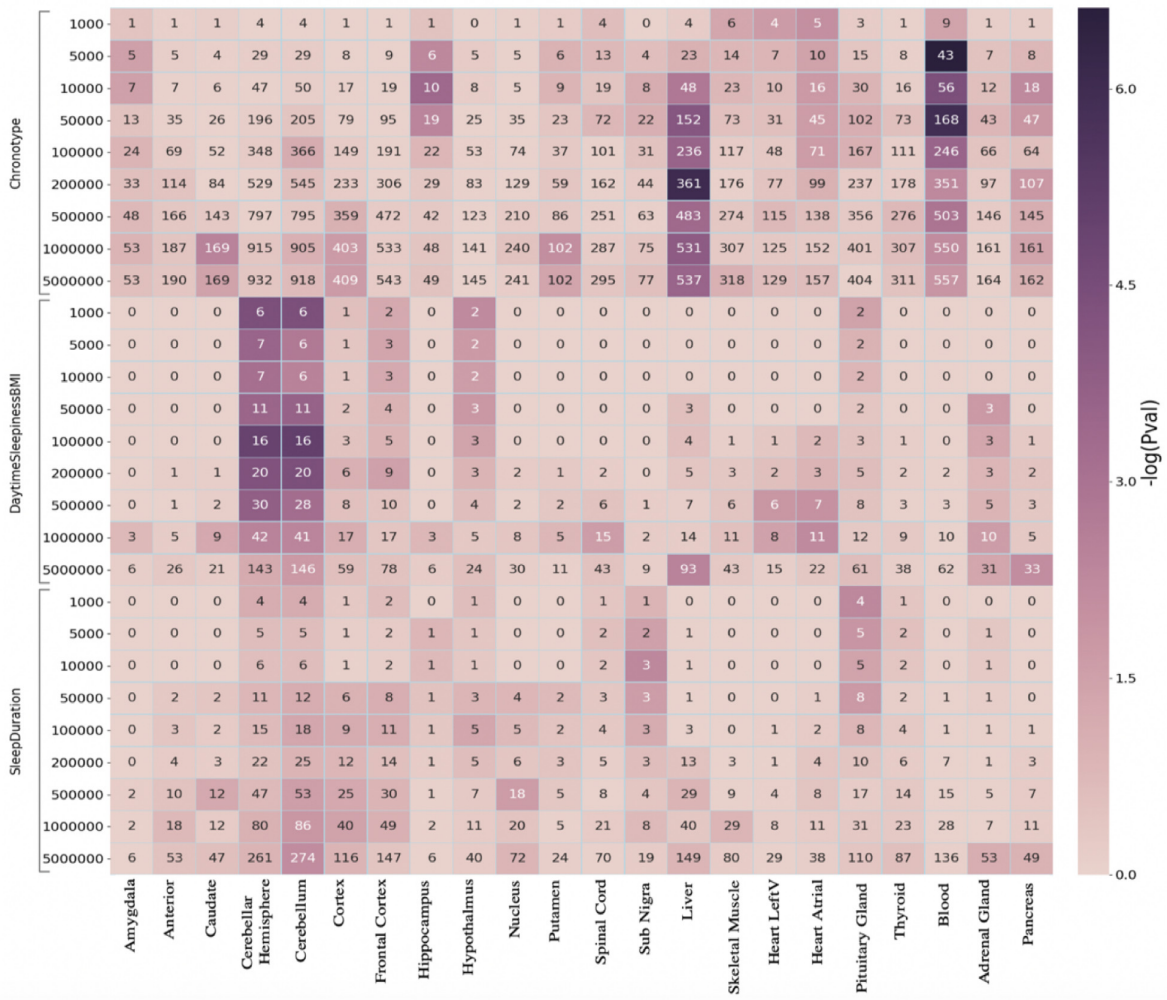
Enrichment of sleep traits SNPs in tissue-specific gene lists (GTEx). The x-axis shows different tissue-specific gene lists, and y-axis shows three sets of sleep related SNPs, expanded to multiple genomic distances. The colour of the cells show − *log*_10_ of p-value of enrichment of corresponding gene list (x-axis) in the genes identified through overlap with expanded SNP intervals, the numbers in the cells show the common genes among the two (used in the calculation of Hypergeometric p-value)

In Figure 3, we show enrichment of SNPs from three sleep related phenotypes, namely chronotype, daytime sleepiness adjusted for BMI, and sleep duration. These enrichments are calculated for tissue-specific genes lists created from GTEx for 22 tissues, the majority of them from the brain. Application of PEGS to these data reveals some strong associations, e.g. chronotype SNPs strongly enriched for genes expressed in liver and blood, while daytime sleepiness SNPs are enriched in gene sets for different brain tissues. Some of these associations are reported in the literature, e.g. daytime sleepiness SNPs in brain tissue ^12^, others may warrant further investigation.

## Conclusions

Through the three different applications above, we demonstrate that PEGS is a versatile and highly efficient tool to integrate different genomic data, and is able to generate hypotheses for further analysis. The implementation of PEGS is highly efficient and as an example of computational efficiency, with pre-created reference TSS files, it only took 7.6 seconds to produce the output for Figure 2A on a laptop with Intel(R) Core(TM) i5-7200U CPU @ 2.50GHz processor with 16GB RAM.

Furthermore, the user can adjust the background population and control for bias. For example, depending on the scientific question at hand, the reference gene interval file (TSSs BED file) could be limited to include only those genes known to be expressed in the tissue of interest. The efficiency of PEGS allows multiple gene and peak input files (e.g. with varying false discovery rate or fold-change cut-offs) to be tested quickly.

PEGS builds on some aspects of, and is complementary to, GREAT ^3^, an existing tool, which performs functional enrichment of regulatory regions using annotations of nearby genes. PEGS could also be used in conjunction with other tools to gain further mechanistic understanding (e.g. by finding enriched transcription factors with TFEA.ChIP ^13^, ranking of their target genes with Cistrome-GO ^14^ or BETA ^15^, or predicting which TFs might regulate differentially expressed gene sets with Lisa ^16^).

## Data availability

All data underlying the results are available as part of the article or available publicly.

## Software availability

Software available from Zenodo: https://zenodo.org/record/5012058\#.YNG7xmhKiUl. It is easily installable through the Python Package Index (PyPI).

Source code available from Github: https://github.com/fls-bioinformatics-core/pegs.

Archived source code at the time of publication: https://doi.org/10.5281/zenodo.5012058 ^1^

License: PEGS is distributed under BSD 3-Clause license.

Online manual: https://pegs.readthedocs.io
