# Supplementary material for: PEGS: An efficient tool for gene set enrichment within defined sets of genomic intervals
Source: F1000Res. 2021 Nov 2;10:ISCB Comm J-570. Originally published 2021 Jul 15. [Version 2] doi: 10.12688/f1000research.53926.2 (PMC8406447; doi:10.12688/f1000research.53926.2)
Supplement: Peer review history

# Peer review: Reviewer response for version 2

**DOI:** 10.5256/f1000research.78711.r98741

**Published:** 2022-02-25

**Authors:** Aziz Khan

**Affiliations:** 1 Stanford Cancer Institute, Stanford University, Stanford, CA, USA

The authors addressed all of my concerns/suggestions in the revised version.

Are the conclusions about the tool and its performance adequately supported by the findings presented in the article?

Yes

Is the rationale for developing the new software tool clearly explained?

Yes

Is the description of the software tool technically sound?

Yes

Are sufficient details of the code, methods and analysis (if applicable) provided to allow replication of the software development and its use by others?

Yes

Is sufficient information provided to allow interpretation of the expected output datasets and any results generated using the tool?

Yes

Reviewer Expertise:

bioinformatics, gene regulation, regulatory genomics, epigenomics

I confirm that I have read this submission and believe that I have an appropriate level of expertise to confirm that it is of an acceptable scientific standard.

# Peer review: Reviewer response for version 2

**DOI:** 10.5256/f1000research.78711.r98740

**Published:** 2021-11-05

**Authors:** Nicolae Radu Zabet

**Affiliations:** 1 Blizard Institute, Barts and The London School of Medicine and Dentistry, Queen Mary University of London, London, E1 2AT, UK

The authors have addressed all my comments.

Are the conclusions about the tool and its performance adequately supported by the findings presented in the article?

Yes

Is the rationale for developing the new software tool clearly explained?

Yes

Is the description of the software tool technically sound?

Yes

Are sufficient details of the code, methods and analysis (if applicable) provided to allow replication of the software development and its use by others?

Yes

Is sufficient information provided to allow interpretation of the expected output datasets and any results generated using the tool?

Yes

Reviewer Expertise:

Computational biology, bioinformatics, chromatin and epigenetics

I confirm that I have read this submission and believe that I have an appropriate level of expertise to confirm that it is of an acceptable scientific standard.

# Peer review: Reviewer response for version 1

**DOI:** 10.5256/f1000research.57359.r89907

**Published:** 2021-08-26

**Authors:** Aziz Khan

**Affiliations:** 1 Stanford Cancer Institute, Stanford University, Stanford, CA, USA

In this paper, the authors presented a Python-based command-line tool, PEGS, for gene set enrichment in association with genomic regions. PEGS computes the enrichment of gene sets with proximity-based association with genomic region sets. These associations are further restricted within the Topologically Associated Domains (TADs), which is good. The manuscript is moderately written and it provides three use cases of the tool.

 The tool itself is very useful but it lacks several key options to give users the flexibility to customize the input data and also the output heatmap.

 I have the following comments for the authors to address:

1. It is useful to restrict peak-gene association within the TAD boundaries, but it is not the case that all the interactions, such as enhancer-gene interactions occur within the TAD boundaries. The enhancer–gene communication can also occur outside topological domains or in-between TADs. Do authors plan to provide an optional feature to integrate chromatin interaction data, such as HI-C?
2. The command-line tool can be further improved by providing additional options to improve user experience and its usage. Below are some recommendations.
  - Currently, the peaks sets and gene lists inputs arguments are positional and the tools can only scan files available in the provided folders. Instead of looking into provided folders for BEDs files and gene lists, the argument should also allow chaining a list of bed files with a path. This is because in real analysis scenarios BEDs can be spread across multiple folders or a single folder can have other visible/hidden files. For example, I was testing the tool on a Mac machine, and PEGS started processing peaks for *.DS_Store*, which is the default directory structure and a hidden file. 
  - The tool arguments could be: pegs --peaks peaks/*.bed --genes genes/*.txt  and also *pegs --peaks A.bed B.bed --genes A.txt B.txt*
  - The output heatmap should also have an option to generate vector-based plots, such as PDF or SVG.
  - Authors may consider adding additional options to adjust the heatmap, such as setting labels, dimensions, colors, and gene/peak set names.
3. Figures can be further improved.
4. Please highlight the limitations of the tool such as the enhancer–gene associations are solely based on proximity.
5. Providing an installation option through Conda using the bioconda channel (https://bioconda.github.io/) will be useful and it will increase the usage/availability of the tool.

Are the conclusions about the tool and its performance adequately supported by the findings presented in the article?

Yes

Is the rationale for developing the new software tool clearly explained?

Yes

Is the description of the software tool technically sound?

Yes

Are sufficient details of the code, methods and analysis (if applicable) provided to allow replication of the software development and its use by others?

Yes

Is sufficient information provided to allow interpretation of the expected output datasets and any results generated using the tool?

Yes

Reviewer Expertise:

bioinformatics, gene regulation, regulatory genomics, epigenomics

I confirm that I have read this submission and believe that I have an appropriate level of expertise to confirm that it is of an acceptable scientific standard.

# Response

**Published:** 2021-10-22

**Authors:** Mudassar Iqbal

**Affiliations:** University of Manchester, UK

We thank the reviewer for constructive review and useful suggestions. We have updated the software taking into account the reviewer’s suggestions, improved the manuscript overall and added more text. Here we will address their individual comments.

*1 - It is useful to restrict peak-gene association within the TAD boundaries, but it is not the case that all the interactions, such as enhancer-gene interactions occur within the TAD boundaries. The enhancer–gene communication can also occur outside topological domains or in-between TADs. Do authors plan to provide an optional feature to integrate chromatin interaction data, such as HI-C?*

 We would like to emphasise that the genomic distances are not restricted to TAD boundaries. PEGS provides the user with the option of supplying any distances. In addition, we also provide the user with the option to restrict peak expansion to TADs boundaries (if available), as a separate analysis (Fig 2A). We have revised the relevant text and we hope this will address reviewer’s main concerns and clarify any confusion. Integration of HiC data is beyond the scope of this work, but we will think of ways to incorporate that in future developments of PEGS.

*2 - The command-line tool can be further improved by providing additional options to improve user experience and its usage. Below are some recommendations.*

- *Currently, the peaks sets and gene lists inputs arguments are positional and the tools can only scan files available in the provided folders. Instead of looking into provided folders for BEDs files and gene lists, the argument should also allow chaining a list of bed files with a path. This is because in real analysis scenarios BEDs can be spread across multiple folders or a single folder can have other visible/hidden files. For example, I was testing the tool on a Mac machine, and PEGS started processing peaks for .DS_Store, which is the default directory structure and a hidden file. *
- The tool arguments could be: pegs --peaks peaks/*.bed --genes genes/*.txt and also pegs --peaks A.bed B.bed --genes A.txt B.txt
- *The output heatmap should also have an option to generate vector-based plots, such as PDF or SVG.*
- *Authors may consider adding additional options to adjust the heatmap, such as setting labels, dimensions, colors, and gene/peak set names.*

We thank the reviewer as these are very useful suggestions and we have updated PEGS to a new version (please see latest version 0.6.2), which includes all of the above command line options. We also output the heatmap data as an excel file, so the user can customise their heatmaps/plots according to their choice/requirements. We have updated the documentation accordingly.

*3 - Figures can be further improved.*

 We have improved and updated all of the figures (please see new version of the manuscript)

*4 - Please highlight the limitations of the tool such as the enhancer–gene associations are solely based on proximity.*

 We have updated the manuscript text making it clear that our enrichment calculations are based on genomic proximity, expanding peaks (in both directions) with given distances (and/or TADs), and obtaining genes whose TSSs overlap with the expanded peaks.

*5 - Providing an installation option through Conda using the bioconda channel (https://bioconda.github.io/) will be useful and it will increase the usage/availability of the tool.*

 We thank the reviewer; our tool is now installable through Conda.

# Peer review: Reviewer response for version 1

**DOI:** 10.5256/f1000research.57359.r89906

**Published:** 2021-08-02

**Authors:** Nicolae Radu Zabet

**Affiliations:** 1 Blizard Institute, Barts and The London School of Medicine and Dentistry, Queen Mary University of London, London, E1 2AT, UK

Briggs and co-authors present a new tool called PEGS to generate gene set enrichment for ChIP-seq and DNase-seq datasets. In fact, the tool can be applied to any set of genomic intervals, including SNPs datasets. Generation of gene set enrichment for genomic intervals is a very important task and the authors propose an interesting approach to address it. Particularly, I appreciate the use of TADs to limit the expansion of genomic intervals. They also provide three use cases with different datasets and prove the applicability of this tool.

 I have the following comments:

1. Do you consider alternative TSS? Would a gene with multiple TSSs be overrepresented or not?
2. Do you think that distal loops connecting TSS with enhancers residing outside of TADs would affect your results?
3. While readable, the resolution of figure 2 is low. I would advise the authors to upload a higher resolution figure.
4. For case1, maybe I missed it, but I think it would be interesting to interpret the results with or without TADs. This would allow us to see the impact of TAD annotation on the analysis.
5. I think the authors should add more explanations in the text about the results of their three cases.

Are the conclusions about the tool and its performance adequately supported by the findings presented in the article?

Yes

Is the rationale for developing the new software tool clearly explained?

Yes

Is the description of the software tool technically sound?

Yes

Are sufficient details of the code, methods and analysis (if applicable) provided to allow replication of the software development and its use by others?

Yes

Is sufficient information provided to allow interpretation of the expected output datasets and any results generated using the tool?

Yes

Reviewer Expertise:

Computational biology, bioinformatics, chromatin and epigenetics

I confirm that I have read this submission and believe that I have an appropriate level of expertise to confirm that it is of an acceptable scientific standard.

# Response

**Published:** 2021-10-22

**Authors:** Mudassar Iqbal

**Affiliations:** University of Manchester, UK

We thank the reviewer for constructive comments, here we will address their points one by one.

*1 - Do you consider alternative TSS? Would a gene with multiple TSSs be overrepresented or not?*

 We consider all TSSs defined in the given genome build, which can include multiple TSSs for some genes. When calculating the enrichment for gene sets obtained through overlap of TSSs with expanded peaks, we remove duplicates. Therefore, genes with multiple TSSs are not over-represented.

*2 - Do you think that distal loops connecting TSS with enhancers residing outside of TADs would affect your results?*

 We have two scenarios for enrichment calculations in PEGS. First, the user can provide genomic distances which are not constrained to TADs. Hence enhancers residing in a separate TAD to the TSS could be included. Secondly, we provide an option to the user to constrain the peak expansion to TAD/subTAD boundaries, if available. This will exclude enhancers outside the TAD boundaries, but the distances option can still be utilised to test multiple distances within and beyond TAD boundaries.

*3 - While readable, the resolution of figure 2 is low. I would advise the authors to upload a higher resolution figure.*

 We agree, and have added a high-resolution version of Fig. 2

*4 - For case1, maybe I missed it, but I think it would be interesting to interpret the results with or without TADs. This would allow us to see the impact of TAD annotation on the analysis.*

 This is related to point 2. In Fig.2A, we do provide the analysis with and without TADs for case 1. Enrichment for multiple distances is at the top two panels, and enrichment calculations using TADs are at the bottom. We have improved Fig 2 to make this clear. 

*5 - I think the authors should add more explanations in the text about the results of their three cases.*

 We have added more text in the manuscript, further explaining the three cases.
